# Recent Advances in Light-Controlled Activation of Pt(IV) Prodrugs

**DOI:** 10.3390/ijms232314511

**Published:** 2022-11-22

**Authors:** Daniil Spector, Kirill Pavlov, Elena Beloglazkina, Olga Krasnovskaya

**Affiliations:** 1 Chemistry Department, Lomonosov Moscow State University, Leninskie Gory 1,3, 119991 Moscow, Russia; 2 Department of Materials Science of Semiconductors and Dielectrics, National University of Science and Technology (MISIS), Leninskiy Prospect 4, 101000 Moscow, Russia

**Keywords:** photoactivatable platinum prodrugs, photodynamic therapy, photocontrolled chemotherapeutics, photocatalysis

## Abstract

Pt(IV) prodrugs remain one of the most promising alternatives to conventional Pt(II) therapy due to their versatility in axial ligand choice and delayed mode of action. Selective activation from an external source is especially attractive due to the opportunity to control the activity of an antitumor drug in space and time and avoid damage to normal tissues. In this review, we discuss recent advances in photoabsorber-mediated photocontrollable activation of Pt(IV) prodrugs. Two main approaches developed are the focus of the review. The first one is the photocatalytic strategy based on the flavin derivatives that are not covalently bound to the Pt(IV) substrate. The second one is the conjugation of photoactive molecules with the Pt(II) drug via axial position, yielding dual-action Pt(IV) molecules capable of the controllable release of Pt(II) cytotoxic agents. Thus, Pt(IV) prodrugs with a light-controlled mode of activation are non-toxic in the absence of light, but show high antiproliferative activity when irradiated. The susceptibility of Pt(IV) prodrugs to photoreduction, photoactivation mechanisms, and biological activity is considered in this review.

## 1. Introduction

Since the discovery of the antiproliferative properties of cisplatin in the 1960s, Pt-based drugs have been on the frontline of antitumor chemotherapy [1,2,3,4]. Platinum-based antitumor drugs are a part of almost 50% of all clinically used chemotherapy regimens [5]. Conventional Pt(II) drugs, including FDA-approved cisplatin, oxaliplatin, and carboplatin, share a similar mechanism of action against tumor cells. Inside the cell, the equatorial chloride or carboxylate ligands are exchanged for water and the aquated Pt(II) complex binds to the N7 position of purine bases, forming crosslinks with DNA, mainly 1,2- and 1,3-intrastrand crosslinks [6,7]. The induced DNA damage and oxidative stress activate apoptosis, which leads to cell death [8].

Despite widespread use in clinical practice, Pt(II) drugs possess critical drawbacks that limit the efficiency of antitumor therapy [9,10]. Due to non-specific binding to biomolecules, all three FDA-approved platinum drugs exhibit acute toxicity to various organs, including nephrotoxicity [11], ototoxicity [12], and neurotoxicity [13]. Another crucial side effect is acquired resistance to platinum drugs during therapy, which decreases the efficacy of Pt(II) antitumor agents by limiting platinum accumulation in cells or deactivating drugs intracellularly [14,15].

To overcome those drawbacks, a variety of different approaches have been proposed, including novel equatorial ligands for cisplatin-like Pt(II) complexes, unconventional trans-platin compounds, and Pt(IV) prodrugs [16]. Pt(IV) prodrugs are octahedral low-spin d^6^ complexes that are less susceptible to equatorial ligand substitution and, consequently, to reactions with biomolecules in the bloodstream [17]. Pt(IV) complexes themselves do not exhibit significant cytotoxicity due to their inability to bind DNA [18]; however, in the presence of biological reductants, such as sodium ascorbate, glutathione, or cysteine, Pt(IV) complexes release their axial ligands and the initial Pt(II) complex [19]. The biological activity of Pt(IV) prodrugs can be finely tuned by varying the nature of axial ligands. To this date, a large number of Pt(IV) prodrugs have been reported that combat resistance of cancer cells to cisplatin [20], have elevated antiproliferative activity due to the enhanced lipophilicity or additional mechanism of toxicity [21,22], lower the severity of platinum drug therapy [23], or demonstrate improved selectivity towards cancer cells [24]. However, despite the substantial progress made in the design of efficient Pt(IV) prodrugs, the complexes often lack selectivity towards cancer cells and therapeutic efficiency in vivo, and are prone to hydrolysis in the bloodstream [25,26,27].

Design of Pt(IV) prodrugs with the controllable mode of release is a strategy that utilizes the advantages of the Pt(IV) prodrug approach while also overcoming the drawbacks, such as low selectivity towards malignant cells. Among various ways to govern the antiproliferative action of compounds, light offers unique controllability due to its non-invasiveness, high precision of applied dosage, and ability to localize the effect [28]. Thus, light is an ideal tool to induce the reduction of Pt(IV) prodrugs. Light is also used in photodynamic therapy (PDT), in which otherwise non-toxic compounds are irradiated to produce cytotoxic short-lived reactive oxygen species (ROS) capable of killing cancer cells [29,30]. Various classes of compounds could be utilized as PDT agents, including flavins [31], porphyrins [32], cyanine [33], and bodipy dyes [34]. Self-assembling nanoparticles based on 3,4-bis(4-(diphenylamino)phenyl)acenaphtho [1,2-b]pyrazine-8,9-dicarbonitrile showed striking antitumor activity in near-infrared (NIR) two-photon excitation mode [35]. Other classes of compounds, such as nucleic acids, could be used as light-controlled drug delivery systems [36].

Several strategies to activate Pt(IV) prodrugs by NIR and visible light have been attempted [37]. The first generation of photoactivated Pt(IV) complexes were diiodo Pt(IV) prodrugs, which showed elevated toxicity and an increase in DNA binding ability under white light irradiation. However, the non-negligible dark toxicity led to the discontinuation of further development of this class of Pt(IV) complexes [38,39,40]. Diazido-Pt(IV) complexes were suggested as a more efficient alternative [41]. Although several prodrugs showed a striking increase in toxicity under irradiation, long exposure for up to 6 h to short-wavelength (<400 nm) light was required to achieve full conversion [29,42]. Light in this wavelength range has an effective penetration depth in human tissues of no more than several micrometers and is thus not effective in real tumors [29,42].

An alternative approach to the design of photoactivated Pt(IV) prodrugs is to use photoactive molecules that facilitate the electron transfer from the reducing agent to the Pt(IV) center. First, the photoabsorber and the Pt(IV) prodrug could be used as a combination of two molecules. In this case, a photoabsorber acts as a photocatalyst that is reduced from its excited singlet or triplet state and then reacts with the Pt(IV) complex. A combination of Pt(IV) prodrugs and flavins was studied in the series of reports by Salassa et al. and is considered in the first section of this review. The potential of rhodamine B to serve as a photocatalyst of Pt(IV) prodrugs reduction was investigated by Deng et al. and is also considered in this part. The second approach is to conjugate a photoabsorber and a Pt(IV) complex in one molecule, which can overcome such drawbacks of the combinational approach as different bioavailability and metabolism of drugs used. Moreover, the speed of the Pt(IV) prodrugs photoreduction process also increases due to the intramolecular step of electron transfer from the photoactive ligand to the Pt(IV) center. All Pt(IV) prodrugs with photoactive molecules in the axial position are discussed in the second part of this review. In addition, the photoabsorbers that are utilized to reduce Pt(IV) prodrugs in the presence of light could also act as PDT agents, thus combining the advantages of PDT and PACT in one approach [43].

The main focus of this review is an investigation of biological activity and Pt(IV) prodrugs photoreduction mechanisms. Recently, several brilliant reviews focused on photoactivable Pt(IV) prodrugs have been published [44,45,46,47,48]. The present review discusses the latest advances in the field and thoroughly examines the most successful approaches to the development of effective antitumor photoactivated prodrugs. To the best of our knowledge, this is the most detailed review devoted to the photoabsorber-mediated Pt(IV) prodrugs photoreduction.

## 2. Light-Controlled Activation of Pt(IV) Prodrugs

### 2.1. Photoinduced Catalytical Reduction of Mixtures of Pt(IV) Prodrugs

Flavins are a group of biomolecules that play a key role in aerobic metabolism, embryonic development, and programmable cell death [49]. Due to the extraordinary redox properties of riboflavin, it is widely used as a photocatalyst in the synthesis of different organic compounds, including thiobenzanilides, coumarins, and nitriles [50].

**Figure 1:**
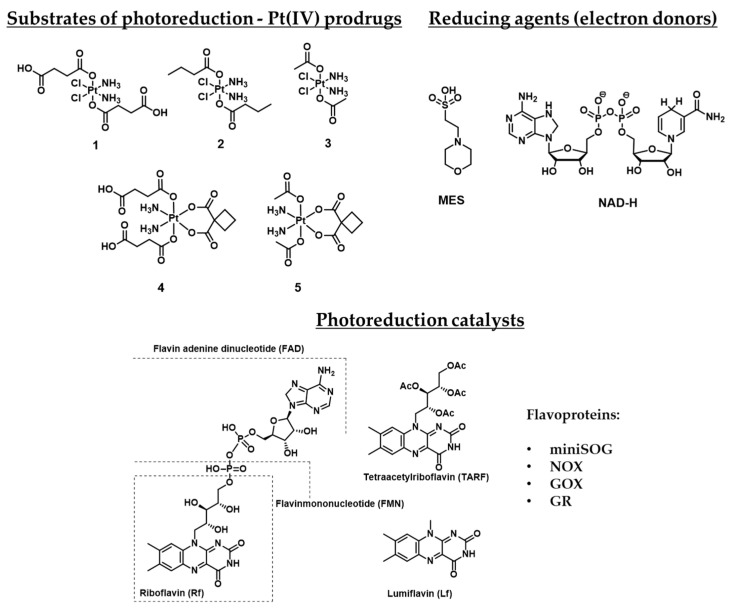
Substrates of photoreduction cisplatin- and carboplatin-based Pt(IV) prodrugs **1–5**, electron donors, and photoreduction catalysts flavins and flavoproteins. Flavoproteins used in the photocatalytic studies: miniSOG (mini singlet oxygen generator, NOX (NADH oxidase), GOX (glucose oxidase), GR (glutathione reductase).

In a series of reports, the scientific group of L. Salassa designed and investigated a combinational approach of Pt(IV) prodrugs photoactivation. Cisplatin-based Pt(IV) prodrugs **1**–**5** that are stable towards hydrolysis in the dark were used as unconventional substrates for photoreduction. Five small-molecule flavin derivatives, including riboflavin Rf, flavin adenine dinucleotide FAD, flavin mononucleotide FMN, tetraacetyl riboflavin TARF, and lumiflavin Lf, were studied as photocatalysts, along with flavoproteins miniSOG (mini singlet oxygen generator), NOX (NADH oxidase), GOX (glucose oxidase) and GR (glutathione reductase). The results are summarized in Table 1 and Figure 1.

The first example of photocatalytic activation of Pt(IV) prodrugs by flavins was reported in 2017 by Alonso-de Castro et al., who demonstrated that cisplatin-based Pt(IV) prodrug **1** with two moieties of succinic acid as axial ligands can be reduced by riboflavin in the presence of MES-buffer under visible light irradiation [51]. Under 5 min of low-dose irradiation (460 nm, 0.75 mW/cm^2^) of a mixture of Pt(IV) prodrug **1** and riboflavin in MES buffer demonstrated that all Pt(IV) prodrug was reduced to cisplatin (Figure 1). Pt(IV) prodrug reduction was confirmed by ^1^H NMR by the disappearance of triplet peaks at 2.3 and 2.55 ppm and the appearance of a singlet peak at 2.35 ppm corresponding to free succinic acid.

**Figure 2:**
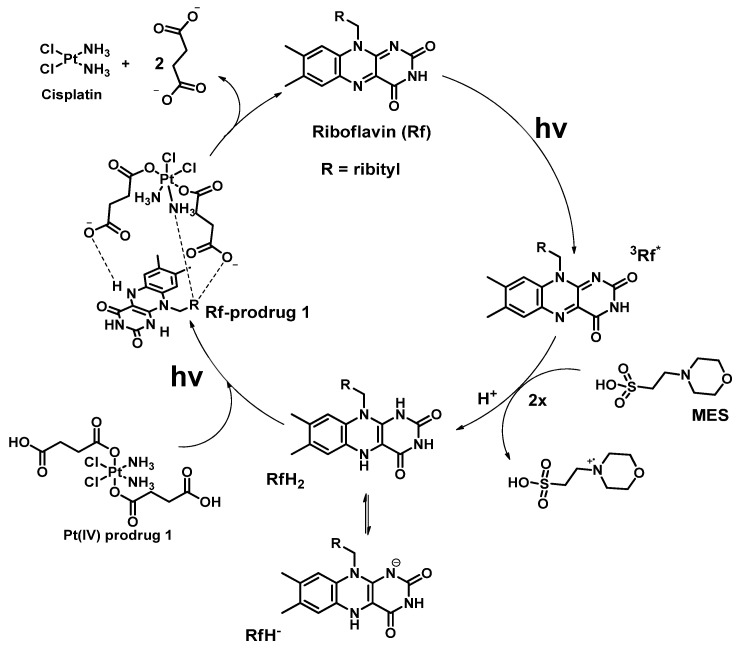
Proposed mechanism of photocatalytic reduction of Pt(IV) prodrug 1 by riboflavin (Rf). The mechanism was proposed based on density functional theory and includes the stages of riboflavin excitation, its reduction to RfH- form, conjugation with Pt(IV) prodrug 1 by hydrogen bonding, and further reductive electron transfer to Pt(IV) prodrug core with the formation of cisplatin and initial riboflavin.

The active catalyst particle was determined by measuring riboflavin fluorescence lifetime in the MES buffer and in the presence or absence of Pt(IV) prodrug. Measurement of riboflavin excited state lifetime using time-correlated single photon counting (TCSPC) ruled out riboflavin excited state as an active catalyst. The redox potential of the reduced form RfH_2_ (approximately −0.2 V) was not sufficient to reduce the prodrug **1** (redox potential −0.9 V). Density functional theory (DFT) modeling of the photocatalytic reduction mechanism showed that Pt(IV) prodrug **1** could form an adduct with RfH_2_ via H-bonds. This intermediate then absorbs the photon and reduces the Pt(IV) prodrug **1** with the elimination of the axial ligands (Figure 2).

The major advantage of PACT is a drastic difference in chemotherapeutic agent cytotoxicity in the dark and under irradiation. To demonstrate this effect, the antiproliferative activity of the Rf-**1** mixture was determined by MTT-assay in the presence and absence of irradiation on a prostate cancer PC-3 (prostate cancer) cell line. The Rf-**1** mixture showed low toxicity in the dark and equivalence to cisplatin cytotoxicity when irradiated with low-dose visible light (460 nm, 0.36 J/cm^2^). Thus, for the first time, the photocatalytic activation of Pt(IV) prodrugs by riboflavin was described, and the light-controlled cytotoxic activity of the Rf-**1** mixture on the PC-3 cell line under irradiation was demonstrated.

Flavoproteins are proteins that contain the riboflavin derivatives FAD (flavin adenine dinucleotide) or FMN (flavin mononucleotide). The flavin moiety in those biomolecules could also act as a photocatalyst of Pt(IV) reduction, thus Alonso-de Castro et al. studied photocatalytic activity towards the reduction of Pt(IV) prodrugs of miniSOG (mini singlet oxygen generator), NOX (NADH oxidase), GOX (glucose oxidase), GR (glutathione reductase), as well as FAD and FMN in a series of NMR experiments [52]. Pt(IV) prodrugs **1**, **2**, and **4** based on cisplatin and carboplatin were chosen as model substrates (Figure 1). The GOX and GR proteins were unable to reduce Pt(IV) prodrugs **1** and **2** due to the small protein surface area available to molecules in the solution. Under blue-light irradiation (460 nm, 6 mW/cm^2^) miniSOG and NOX showed significant catalytic activity towards Pt(IV) prodrugs **1** and **2** in the presence of MES. Interestingly, NOX was also able to reduce **1** and **2** prodrugs in the dark in the presence of NADH.

In a further report by Alonso-de Castro et al., the antiproliferative activity of Rf-**1** and Rf-**4** mixtures was assessed on the PDT-resistant Capan-1 cell line (pancreatic cancer) [53]. Both mixtures under low-dose light irradiation (460 nm, 0.36 J/cm^2^) demonstrated higher cytotoxicity than in the dark. To confirm that upon light-induced activation with riboflavin Pt(IV) prodrugs release species that could bind with DNA, the formation of DNA cross-links was studied by circle dichroism (CD) and MALDI-TOF spectra. After incubation of the Rf-**1** mixture with various oligodeoxyribonucleotides (ODNs), intrastrand cross-links were detected using both methods. Photoinduced DNA damage was studied in Capan-1 cells treated with Rf-**1** mixtures and irradiated with low-dose light (460 nm, 0.36 J/cm^2^) by immune-blotting assay and immunofluorescence microscopy. Significantly increased hyperphosphorylation of the DNA-damage repair-initiating histone H_2_AX in irradiated cells compared to those incubated with Rf-**1** in the dark was a clear marker of light-dependent DNA damage.

An alternative approach to photoreduction of Pt(IV) prodrugs was shown by Mazzei et al. [54]. FMN was loaded into the Au nanoparticles modified by a C11 thiol linker with terminal triazacyclononane (TACN) moieties. As a result, a supramolecular nanozyme was obtained that photocatalyzed the reduction of the Pt(IV) prodrug **1**. In the absence of light, FMN-modified Au nanoparticles demonstrated 15% of Pt(IV) **1** reduction independently from FMN presence, whereas after 30 min of irradiation prodrug by low-dose visible light (460 nm, 5.3 mW/cm^2^) in presence of FMN conversion was 90%.

Gurruchaga-Pereda J. et al. [55] showed the ability of different riboflavin derivatives, namely Rf, FMN, TARF, Lf, and flavoprotein miniSOG, to photoreduce Pt(IV) prodrugs **1**, **3**–**5**. Flash photolysis and TCSPC techniques were used to gain insight into the role of different excited states in the catalytic process. Flash photolysis (or transient absorption spectroscopy) is a pump-probe technique that allows studying the kinetics of unstable intermediates that are generated from pump laser pulse by measuring the difference in absorption on a scale from femtoseconds to micro and milliseconds [57].

To identify the most efficient flavin-based catalyst, Pt(IV) prodrug-flavin parameters TOF (turnover frequency) and TON (turnover number) were calculated for each pair by ^1^H NMR study. For TARF and FMN the highest TOF numbers (≈20) were observed, while flavoprotein miniSOG showed the lowest TOF values of less than 6.

The main hypothesis of the flavin-catalyzed photoreduction mechanism was that FMNH^−^ is formed from the reaction of FMN triplet excited state with electron donors such as NADH. To confirm the hypothesis, TCSPC and flash photolysis were utilized. TCSPC assay showed that the singlet excited state of flavin does not participate in the formation of a reducing catalytic particle RfH^−^ as fluorescence lifetime was not altered by the presence of NADH. On the other hand, flash photolysis showed that the triplet excited state of riboflavin took part in the formation of FMNH^−^ reduced form as FMN triplet state lifetime was decreased in the presence of NADH. In addition, the role of FMNH^−^ as the active catalytic particle was confirmed by monitoring the evolution of UV-Vis spectra. FMN was first reduced to FMNH^−^ using NADH, which was observed at UV-Vis spectra by the disappearance of FMN bands at 300–500 nm. When the Pt(IV) prodrug **1** was added to the mixture, FMN spectral features were restored, thus confirming that FMNH^−^ reacts with prodrug **1** to produce the Pt(II) complex and FMN.

In the recent paper by Gurruchaga-Pereda J. et al., mutant analogs of miniSOG (Q103V, Q50W, and Q50E) and their catalytic activity toward the reduction of Pt(IV) prodrugs **1**, **3** were investigated [56]. Q103V mutant turned out to be the most efficient catalyst as it required only 90 s of irradiation (460 nm, 6 mW/cm^2^) to fully reduce Pt(IV) prodrugs **1** and **3**, compared to 200–300 s for other mutants and wild-type (WT) protein, which was demonstrated by monitoring the reduction process via NMR.

In the previous work, the role of the triplet excited state of riboflavin derivatives in the photocatalytic reduction of Pt(IV) prodrugs reduction was established. To confirm the relation between the triplet state lifetime of flavoproteins and the speed of Pt(IV) prodrugs photoreduction reaction, the excited triplet state lifetime of WT miniSOG and its mutants in aerated solutions was measured using flash photolysis. The most efficient catalyst, Q103V, had the longest triplet state lifetime of 102 µs, 3–56-fold higher than for other proteins. Thus, Gurruchaga-Pereda et al. showed that site-targeted mutagenesis can improve the catalytic activity of flavoproteins toward Pt(IV) prodrugs substrates by stabilizing the triplet excited state of a protein.

**Figure 3:**
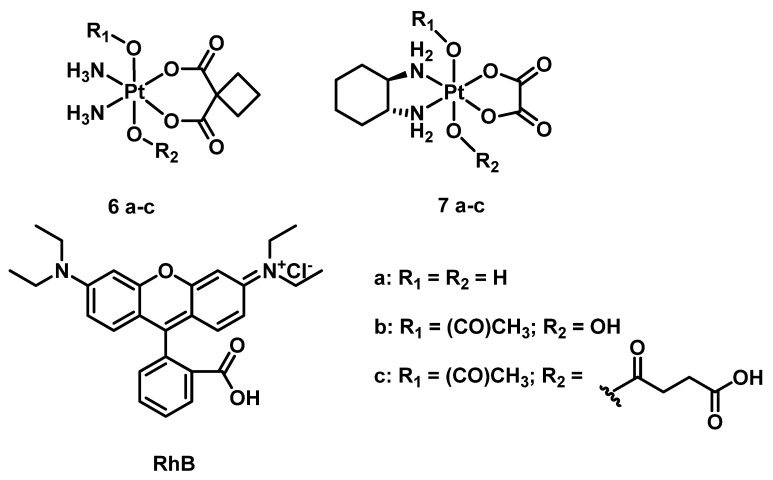
Carboplatin and oxaliplatin-based Pt(IV) prodrugs used as a substrate in rhodamine B-promoted photoreduction studies.

Another class of photoabsorbers was also studied as potential photocatalysts for Pt(IV) prodrugs. The catalytical activity of rhodamine B (RhB) to reduce Pt(IV) prodrugs **6a–c** and **7a–c** was investigated by Deng et al. (Figure 3) [58]. Pt(IV) prodrugs **6a–c** and **7a–c** were stable in the equimolar mixture in the presence of 20 equivalents of sodium ascorbate and in the absence of light. Under white light irradiation (400–760 nm, 4 mW/cm^2^) after 5 h, only 4% of **6b** and 9% of **6c** were reduced to carboplatin, while **6a** was barely reduced. For oxaliplatin-based Pt(IV) prodrugs **7a–c**, less than 10% of the prodrugs **7a** and **7b**, while 60% of **7c**, was reduced to oxaliplatin after 5 h in the same irradiation mode. The slow reaction rate observed prompted the researcher to attempt conjugating RhB and Pt(IV) center covalently, an approach that will be considered in the next part of the review.

In summary, in several reports [51,52,53,54,55,56] Salassa et al. showed that flavins and flavoproteins are efficient catalysts for the reduction of Pt(IV) prodrugs. The small molecules TARF and FMN were the most efficient catalysts with the highest TOF values of about 20. It was established that photocatalyzed reduction of Pt(IV) prodrugs occurs via a reaction of the complex with riboflavin reduced form RfH^−^. The catalytic activity of flavoproteins was also proved to be tuned through selective mutagenesis that stabilizes the excited triplet state of the protein. Rhodamine B was also considered as a catalyst of Pt(IV) prodrugs photoreduction by Zhu et al. [58], but the reaction rate in the span of several hours was too slow for further development of this approach.

A combination of flavins with Pt(IV) under blue-light low-dose irradiation showed cytotoxicity comparable to that of cisplatin on various cell lines, including prostate cancer PC-3 and PDT-resistant CAPAN-1. In the absence of light, toxicity of the flavin-Pt(IV) prodrugs mixtures was negligible, thus showing that photocatalytic light activation localizes the cytotoxic effect of Pt(IV) prodrugs. Controllability over the antiproliferative activity of Pt(IV) prodrugs via flavin-based photocatalysis offers high spatial and temporal selectivity of cytotoxic effect and allows the combination of both PACT and PDT principles in one anticancer treatment strategy.

### 2.2. Photoinduced Reduction of Pt(IV) Prodrugs with Photoabsorbers as Axial Moieties

One of the main advantages of the Pt(IV) prodrug approach is tuning their activity by varying the nature of the axial ligands [16,27,59]. Conjugation of a photoabsorbing molecule with Pt(IV) center opens the path to photocaged Pt(IV) prodrugs and allows overcoming such drawbacks of combinational therapy as different bioavailability, metabolism, and pharmacokinetics of compounds [46]. Moreover, by using a PDT agent as an axial ligand for light-controlled activation of Pt(IV) prodrugs, a dual-action antitumor agent could be obtained that releases Pt(II) species and generates cytotoxic ROS species under light irradiation.

In this section of the review, we discuss 20 Pt(IV) prodrugs with photoactive ligands in the axial position reported to date, the proposed paths of photoactivation, and reported biological activity (Table 2).

#### 2.2.1. Pt(IV) Prodrug with Red-Absorbing Ligand Pyropheophorbide in the Axial Position

**Figure 4:**
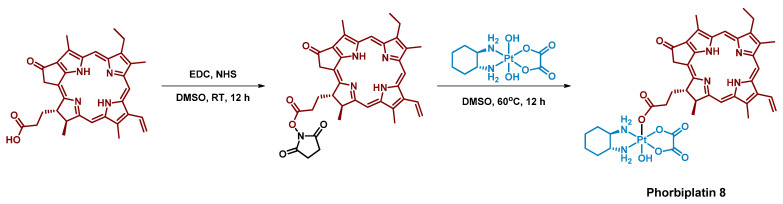
Synthesis of the Pt(IV) prodrug hPhorbiplatin **8** with pyropheophorbide a in axial position.

One of the first Pt(IV) prodrugs conjugated with photoactive ligand pyropheophorbide a (PPA), was reported by Wang et al. [60] (Figure 4). PPA is a photoabsorber with high absorbance at 650 nm and also an efficient singlet oxygen generator [69]. Light in the NIR region of 650–900 nm can penetrate at the highest depth in human tissues, which makes it the optimal region for the design of bioimaging and PDT agents [42]. Thus, a Pt(IV) prodrug with NIR-absorbing ligand could be activated deeply in the tumors, which could increase the efficiency of the therapy.

The stability of Phorbiplatin **8** in PBS buffer and photoactivation kinetics under red light irradiation were monitored using RP-HPLC. The prodrug was stable in the dark in PBS buffer and showed only an 11% reduction after 24 h in the presence of sodium ascorbate. In contrast, under irradiation (650 nm, 7 mW/cm^2^) and with sodium ascorbate in PBS buffer, the Pt(IV) prodrug **8** was reduced within 30 min, thus proving that Phorbiplatin could be activated by red light.

The role of excited singlet and triplet states of Phorbiplatin **8** in the photoreduction mechanism was studied by observing the differences between PPA’s and Phorbiplatin’s spectral features using TCSPC and transient absorption (TA) spectroscopy respectively. Phorbiplatin **8** fluorescence lifetime was the same in the absence and in the presence of the reducing agent, which indicates that the singlet excited state does not participate in the photoreduction process. In the TA spectrum of PPA, a long-lived species of PPA*^−^ with a decay time of 111 µs was detected in the presence of sodium ascorbate [70,71], while for Phorbiplatin **8** in the same conditions the transient state lifetime was only 3 µs, indicating a fast reaction of PPA*^−^ axial ligand with Pt(IV) center.

**Figure 5:**
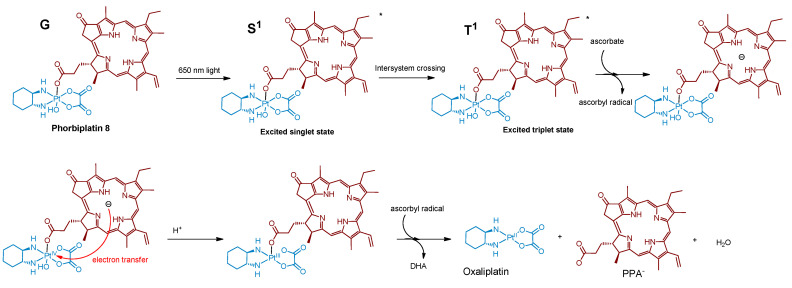
Proposed photoreduction mechanism of Phorbiplatin **8**.

Based on the combined evidence, the photoreduction mechanism of Phorbiplatin **8** was proposed (Figure 5). Phorbiplatin **8** is excited into the singlet state, then the singlet excited phorbiplatin undergoes intersystem crossing into the triplet excited state. In the triplet state, the PPA ligand reacts with the sodium ascorbate, thus generating the ground state PPA radical anion, which then quickly reduces the Pt(IV) core into the Pt(III) via single electron transfer, followed by dissociation of one of the axial ligands. Pt(III) complex then quickly reduces into the Pt(II) oxaliplatin complex.

Phorbiplatin **8** showed little to no toxicity in the dark on various cell lines (Table 3). Under irradiation (15 min, 650 nm, 7 mW/cm^2^) the prodrug showed submicromolar toxicity, with IC_50_ values similar to those of PPA. To confirm that Phorbiplatin **6** photoreduction products could bind genomic DNA, the level of platinum in DNA was measured in cells treated with Phorbiplatin. The platinum level in genomic DNA of irradiated (15 min, 650 nm, 7 mW/cm^2^) A2780 cells was 4-fold more than for cells incubated in the dark.

In vivo study of Phorbiplatin **8** antitumor efficiency on BALB/C mice was conducted on murine mammary adenocarcinoma 4T1 xenograft tumors. Doses of 3.5 µmol Pt/kg of phorbiplatin were intravenously injected five times once in two days and irradiation (660 nm, 100 mW/cm^2^, 10 min) was applied 4 h after injection. After 12 days of therapy, a 67% reduction of tumor volume in the group treated with Phorbiplatin **8** and irradiation compared to the group treated with saline was observed. Interestingly, the antitumor efficiency of the oxaliplatin-PPA mixture and irradiation showed no statistically significant difference from the oxaliplatin and control groups. The change in body weight of mice in all groups was insignificant. Thus, the synergetic effect of oxaliplatin and PPA combination in one prodrug was demonstrated.

#### 2.2.2. Phorbiplatin-Containing Nanoprodrug with NIR Absorption

**Figure 6:**
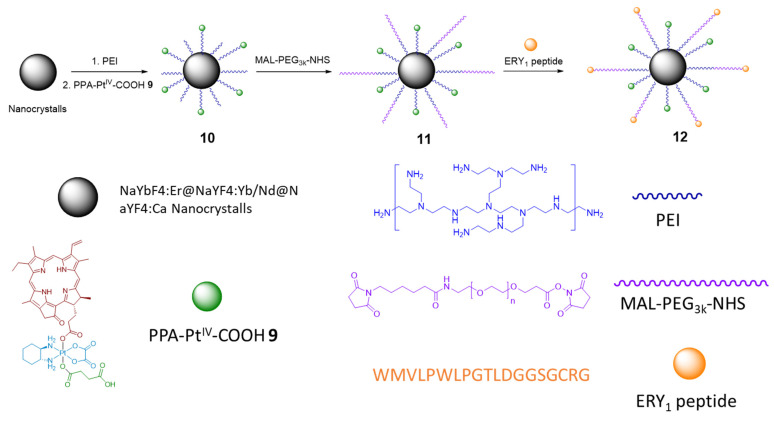
Structure of nanocrystals **10**–**12**, modified with PPA-containing Pt(IV) prodrug **9**.

In the subsequent study, Phorbiplatin **8** prodrug was loaded onto NaYbF4:Er@NaYF4:Yb/ Nd@NaYF4:Ca nanocrystals (NCs) via linkage with amine groups (Figure 6) to increase the Pt(IV) prodrug **8** stability in blood and shift the activation wavelength further in the deeply-penetrating NIR region [61]. Using ESI-MS, Pt(II) release from nanocrystals under NIR irradiation (808 nm) in the presence of ascorbate was confirmed. At this wavelength, NCs **10** generate upconversion luminescence at around 670 nm [72], which then activate Phorbiplatin prodrug **9** on the surface of the nanocrystals and induce the release of initial Pt(II) complex oxaliplatin. The NCs **10** were also found to generate singlet oxygen ^1^O_2_ when irradiated with 808 nm light.

**Figure 7:**
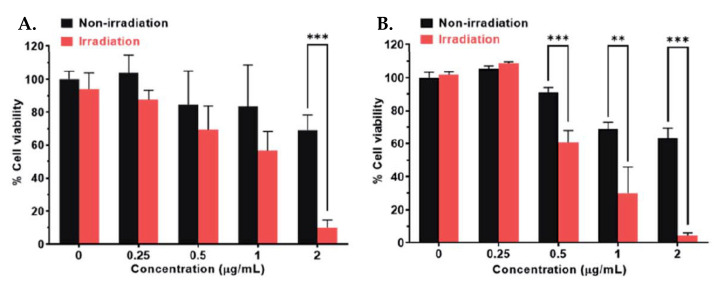
Cytotoxicity of NCs **10** on A2780 (**A**) and A2780cisR (**B**) cell lines. **, *p* < 0.01; ***, *p* < 0.001. Reproduced with permission from Ref. [61]. Copyright 2021 The Royal Society of Chemistry.

Photocytotoxicity of NCs **10** were tested on 4T1 (murine mammary adenocarcinoma), MCF-7 (breast adenocarcinoma), A2780 (ovarian adenocarcinoma), and cisplatin-resistant A2780cisR. After cells were irradiated with NIR light (808 nm, 500 mW/cm^2^, 5 min) at 2 µg/mL their viability significantly decreased compared to cells incubated in the dark. For the A2780cisR cell line, cell viability after irradiation was 4.4% compared to 70% of survived cells in the absence of irradiation (Figure 7). Thus, NCs **10** exhibited antiproliferative properties selectively under NIR irradiation. NCs **10** were also more effective as a drug delivery vehicle as its level of intracellular platinum in A2780cisR cells after 2 h of incubation surpassed one of the Pt(IV) prodrug **9** by 20-fold.

To increase the selectivity of the nanoprodrug **10** towards tumors, Pt-NCs **10** were functionalized with O-[*N*-(6-maleimidohexanoyl)aminoethyl]-O0-[3-(Nsuccinimidyloxy)-3-oxopropyl]polyethylene glycol 3000 (NHS-PEG-MAL) linker yielding NCs **11** and, subsequently, glycophorin A-binding (ERY_1)_ peptide that strongly and specifically bind with the surface of mouse erythrocytes [73], yielding NCs **12** as a result.

The stability of obtained NCs **12** in mouse blood was studied via RP-HPLC with Pt(IV) prodrug **9** and NCs **10** as controls. NCs **12** were quite stable in the blood (91% remained intact after 24 h), while only 71% of NCs **10** and 29% of Pt(IV) prodrug **9** remained after 24 h of incubation. The ability of NCs **11** and **12** to bind erythrocytes was determined using scanning electron microscopy. Almost all NCs **12** were bound to erythrocytes, while the binding efficiency of NCs **9** was only 1%, indicating that NCs **12** efficiently bind with erythrocytes via an ERY_1_ peptide linkage.

Analysis of blood samples via inductively coupled plasma–optical emission spectroscopy (ICP-OES) showed that NCs **12** had a bloodstream circulation half-life of 907 h, while only 0.79 h were observed for the Pt(IV) prodrug **9**. To confirm the hypothesis that the elevated bloodstream circulation time of NCs **12** would lead to elevated accumulation of the compound in the tumor, platinum intratumoral content for Pt(IV) nanoprodrugs **10**–**12** in the 4T1 xenograft tumor model of BALB/C mice was analyzed at different time points by ICP-OES and fluorescence bioimaging. Mice in each group were injected with 1.3 mg Pt/kg of each nanoprodrug **10**–**12**, and fluorescent imaging and samples for the ICP-OES test were obtained at 5 min, 3 h, 17 h, and 24 h. For the Pt(IV) prodrug **9**, NCs **10,** and **11** the amount of platinum in tumors decreased gradually for 24 h after the injection, while for NCs **12** it remained stable for 17 h followed by a slight increase at 24 h.

**Figure 8:**
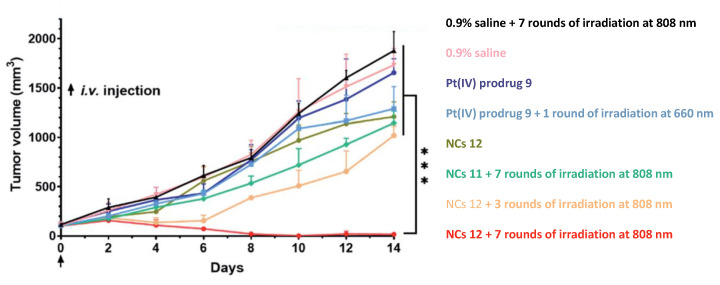
Antitumor activity of complexes **9**, **11**, **12** on 4T1 tumor. Irradiation conditions: 660 nm laser (100 mW cm^−2^) for 10 min per mouse for prodrug **9**, 808 nm laser (0.5 W cm^−2^) for 30 min (5 min irradiation with 5 min intervals) per mouse for NCs **11** and **12.** ***, *p* < 0.001 Reproduced with permission from [61]. Copyright 2021 The Royal Society of Chemistry.

The antitumor efficiency of NCs **12** was examined on a 4T1 tumor model in BALB/C mice with saline, the Pt(IV) prodrug **9,** and NCs **11** used as control groups. Mice were injected once with doses of 2.5 µmol Pt/kg for each complex (Figure 8). For 7 days, the tumors of mice in the NCs **12** group were irradiated with 808 nm light (500 mW/cm^2^, 5 min of irradiation with 5 min interval, 30 min total). Striking antitumor efficiency of NCs **12** with peptide vector was observed after 14 days of therapy, with the average tumor volume only 16.1 mm^3^ for NCs **12**, more than 100-fold less than for the group treated with saline (1750 mm^3^) or the Pt(IV) prodrug **9** (≈1600 mm^3^). Moreover, two of the five mice in the group were tumor-free by the end of the experiment. Thus, the unique antitumor properties of the Pt(IV) nanoprodrug **12** modified with an erythrocyte-binding vector were shown by Wang et al. [61].

#### 2.2.3. Pt(IV) Prodrug with Coumarin and Cell-Penetrating Peptide in the Axial Positions

**Figure 9:**
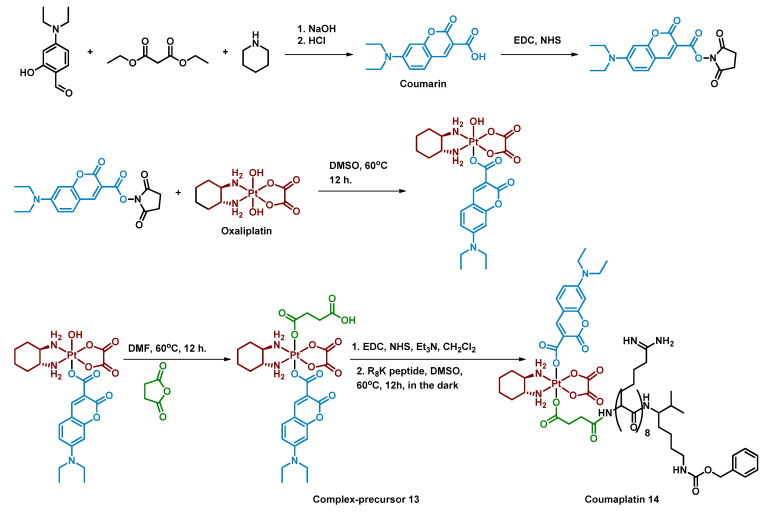
Synthesis of the Pt(IV) prodrug Coumaplatin **14** and its complex-precursor **13** with coumarin in axial position.

**Figure 10:**
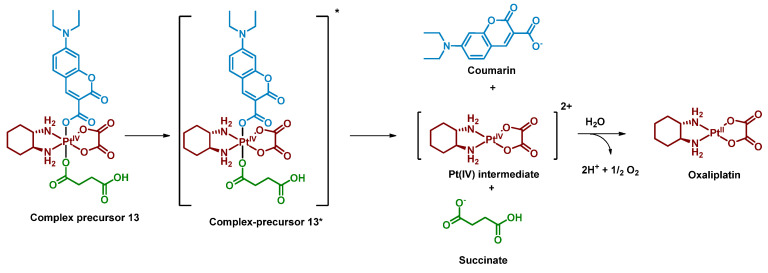
Proposed mechanism of the Pt(IV) prodrug **13** photoreduction.

Deng et al. reported a photocaged Pt(IV) prodrug that can be controllably activated by low-intensity blue light (450 nm, 8 mW/cm^2^) [62]. The controllable activation was achieved by introducing a coumarin moiety into the oxaliplatin axial position (Figure 9). The Pt(IV) prodrug **13** was stable in the phosphate buffer in the dark for 24 h, while only 1.1% of the complex remained after irradiation with blue light (450 nm, 8 mW/cm^2^, 1 h) in the absence of sodium ascorbate. The prodrug **13** was found to be reduced only in protic solvents and the photoreduction of the complex was studied while monitoring the pH of the solution. The slight decrease of the solution’s pH indicated that protons were generated along with the prodrug photoreduction. Thus, water was proposed as the reducing agent of the Pt(IV) complex **13**, which was then proved by detecting ^18^O^16^O oxygen above the solution of the complex in water enriched with H_2_^18^O (Figure 10). The same mechanism was suggested for Coumaplatin **14**.

To improve the ability of the oxaliplatin-coumarin Pt(IV) prodrug **13** to reach the nucleus of cancer cells, the second axial position of the complex **13** was modified by cell-penetrating R_8_K peptide (Figure 8) [74]. The resulting complex Coumaplatin **14** showed significant dark stability in tumor cells, while under irradiation (450 nm, 8 mW/cm^2^, 1 h) it quickly released Pt(II) complex and axial ligands, as was shown via RP-HPLC. Coumaplatin **14** was highly effective in accumulating platinum in the cell nucleus, with more than 86% of platinum localized in the nucleus of A549cisR cells compared to 22% and 7% for oxaliplatin and complex **13**, respectively.

The photocytotoxicity of Coumaplatin **14** was evaluated on various cancer cells (Table 4). The toxicity of Coumaplatin **14** in the dark was comparable to that of oxaliplatin, while after blue-light irradiation (450 nm, 8 mW/cm^2^, 1 h) it increased 7–62-fold. Interestingly, the toxicity of the Pt(IV) prodrug **13** increased only slightly after irradiation, which indicates a crucial impact of nucleus-targeting R_8_K peptide axial ligand in enhancing Coumaplatin **14** cytotoxicity. Notably, the toxicity of Coumaplatin **14** was significantly high in cisplatin-resistant A549cisR and A2780cisR cell lines, indicating the ability of the Pt(IV) prodrug **14** to overcome resistance to cisplatin.

Cytotoxicity tests on 3D cellular cultures showed that Coumaplatin **14**, but not oxaliplatin, accumulated in the necrotic regions of spheroids, which was shown by confocal microscopy. Further studies revealed that senescence was the major factor of cell death rather than apoptosis. Moreover, Coumaplatin **14** was found to trigger an immune response as three major ICD biomarkers (calreticulin exposure, release of high-mobility group box 1 protein, and ATP secretion) were activated in A549cisR cells upon exposure to Coumaplatin **14**; activation of those biomarkers was not observed after incubation with oxaliplatin.

Thus, Coumaplatin **14** is a unique Pt(IV) prodrug that can be activated by low-dose blue light and deliver cytotoxic Pt(II) complex directly into the cell nucleus. The prodrug can be activated even in the absence of reducing agents and its cytotoxicity mechanism differs greatly from that of oxaliplatin, which indicates that the prodrug could overcome resistance to conventional platinum drugs.

#### 2.2.4. Pt(IV) Prodrugs with Rhodamine B in the Axial Position

**Figure 11:**
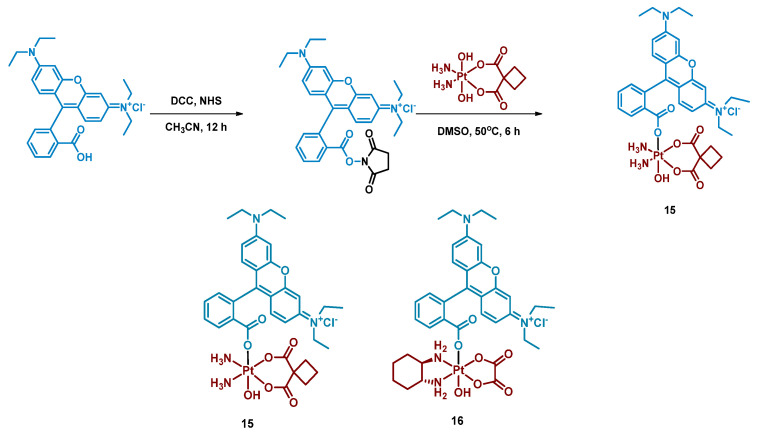
Synthesis of Pt(IV) prodrugs Rhodaplatin **15** and structures of Pt(IV) prodrugs Rhodaplatins **15** and **16** with rhodamine B in axial position.

Rhodamine B is a widely used fluorescent dye that bears an internal photoswitch. It also exhibits the properties of a photocatalyst in several reactions. To exploit those properties, rhodamine B was utilized as an axial ligand of carboplatin and oxaliplatin resulting in Pt(IV) prodrugs rhodaplatins **15** and **16**, respectively (Figure 11) [58]. Photoactivation properties of Rhodaplatins **15** and **16** were monitored by HPLC. Both prodrugs were highly stable in the dark, even in the presence of sodium ascorbate, with 94% of Rhodaplatin **15** and 88% of Rhodaplatin **16** remaining after 24 h of incubation. However, under low-dose irradiation (400–760 nm, 4 mW/cm^2^ ≈ 5 min) in the presence of sodium ascorbate, both prodrugs **15** and **16** quickly released the initial Pt(II) complexes in less than 5 min.

Insight into the photoreduction mechanism was obtained by measuring the quantum yield and fluorescence lifetimes of RhB and Rhodaplatins **15** and **16**. RhB had higher quantum yield than Pt(IV) prodrugs **15**, **16**, (0.34 vs. 0.18 and 0.19) and fluorescence lifetimes (2.0 vs. 1.0 and 1.1 ns). Moreover, the changes in absorption spectra of the RhB and sodium ascorbate mixture under irradiation indicated the formation of a reduced RhB adduct [75]. Thus, the electron transfer from reduced rhodamine ligand to platinum was proposed as the most probable mechanism of Rhodaplatins **15** and **16** photoreduction. Ascorbate was established as an electron donor of photoreduction by detecting ascorbate radicals in an irradiated solution of Rhodaplatin **16** using electron paramagnetic resonance.

The proposed mechanism included excitation of rhodamine ligand and reduction by ascorbate to RhB radical, followed by electron transfer from rhodamine ligand to Pt(IV) center with the formation of Pt(III) intermediate. Then the photoreduction cycle is repeated and Pt(II) complex is released.

Intracellular stability studies of cellular lysates using HPLC showed that more than 90% of rhodaplatins **15**, **16** remained intact after 6 h of incubation in A2780cisR cells. Photocytotoxicity of Pt(IV) prodrugs **15** and **16** was studied on several cell lines, including cisplatin-resistant A549cisR and A2780cisR (Table 5). Both prodrugs demonstrated a 3–7-fold increase in toxicity when cells were irradiated (400–760 nm, 4 mW/cm^2^, 30 min) compared to cells incubated in the dark. Furthermore, under irradiation, Rhodaplatins **15** and **16** were up to 10-fold more active than their corresponding Pt(II) drugs: rhodaplatin **16** showed 9.8-fold lower IC_50_ values on the A2780cisR cell line than oxaliplatin under irradiation.

The intracellular distribution of rhodaplatin **16** in A2780cisR cells was studied using fluorescent staining. The results indicated that most of the complex is accumulated in mitochondria. DNA damage studies further corroborated the hypothesis that mtDNA is the main target of rhodaplatin **16** as no nuclear DNA (nDNA) damage of A2780cisR cells was observed while mtDNA was damaged significantly; more than a 5-fold increase in Pt content in mtDNA was observed for rhodaplatin **16** under irradiation compared to cells incubated in the dark. Therefore, it was proven that Rhodaplatin **16** exhibits its toxicity mechanism through mitochondria targeting and mtDNA damage.

#### 2.2.5. Carboplatin-Based Pt(IV) Prodrug with Bodipy Derivative in the Axial Position

**Figure 12:**
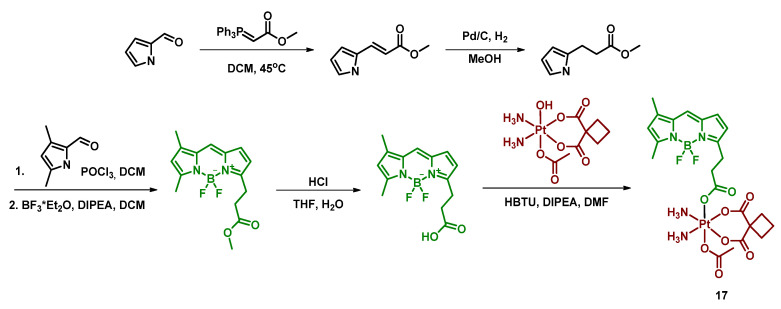
Synthesis of the Pt(IV) prodrug Bodi-Pt **17** with bodipy derivative in axial position.

Boron dipyrromethenes (bodipy) are a class of organoboron fluorophores that are characterized by high fluorescence quantum yields and chemical and photostability [34,76,77]. A prodrug of carboplatin with bodipy **17** was synthesized and studied by Yao et al. (Figure 12). BODI-Pt **17** was found to have a 27-fold lower fluorescence quantum yield than the ligand (0.03 and 0.81, respectively), which authors supposed is due to the heavy atom effect. The complex **17** was also found to be able to generate singlet oxygen in the experiment with singlet oxygen trap 1,3-diphenylisobenzofuran (DPBF), although the efficiency of the ^1^O_2_ production was lower than that of the bodipy ligand.

The Pt(IV) prodrug **17** was found to be stable in the dark, both in the absence and in the presence of sodium ascorbate. In contrast, under irradiation (green light, 13 mW/cm^2^), BODI-PT **17** was reduced even without the reducing agent within 10 min. The studies of BODI-Pt **17** binding with calf thymus DNA (ct-DNA) demonstrated that without irradiation, the level of DNA platination is very low (5.9% of Pt bound to ct-DNA), while after 30 min of irradiation, 30% of the platinum was bound to the ct-DNA.

Cytotoxicity of BODI-Pt **17** was studied on several cell lines, including MCF-7, MDA-MB-231, and A2780 (Table 6). BODI-Pt showed low toxicity in the dark, although surpassing that of carboplatin. However, when irradiated with green light, IC_50_ values of BODI-Pt decreased 2–11 fold compared to those in absence of irradiation. Moreover, the Pt(IV) prodrug **17** showed a 6.5–43-fold increase in toxicity compared to carboplatin under irradiation, which shows significantly improved phototoxicity compared to the initial Pt(II) complex.

Additional experiments were carried out to explain the enhanced cytotoxicity of BODI-Pt **15** compared to carboplatin. BODI-Pt **17** showed increased DNA binding ability in MCF-7 cells, as 0.19 ng of Pt/µg DNA and 0.92 ng of Pt/µg DNA were detected via ICP-MS after 30 min of irradiation and 12 h of incubation for carboplatin and BODI-Pt, respectively. To study the cell death mode induced by the prodrug **17,** confocal images of MCF-7 cells incubated with BODI-Pt **17** and irradiated for 30 min with green light were obtained.

PI and Hoechst 33342 (red and blue staining, respectively) were used to visualize DNA. Blebbing and increased permeability to PI were observed, which indicated that oncosis is the cell death mode of cells incubated with BODI-Pt, but not of cells incubated with carboplatin [78].

In addition, the ability of BODI-Pt to generate cytotoxic ROS intracellularly under green light irradiation was demonstrated using dihydroxyethidium as a ROS detector. Thus, under green light irradiation, BODI-Pt could induce cell death via oncosis through the release of Pt(II) complex that binds with DNA and ROS generation.

#### 2.2.6. Pt(IV) Prodrugs with Linkers of Various Lengths between Pt(IV) Center and Bodipy Ligand

**Figure 13:**
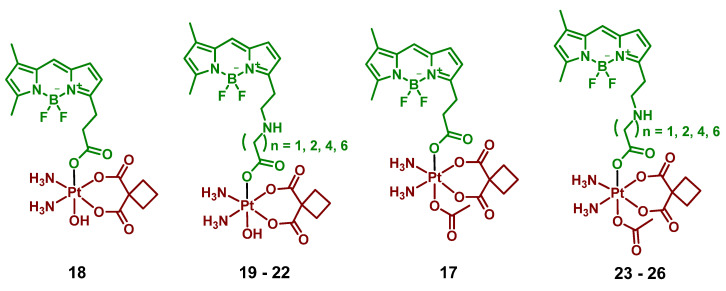
Structures of Pt(IV) prodrugs **17**–**26** with bodipy derivative in the axial position.

In the following report by Yao et al., the length of a linker between the bodipy scaffold and Pt(IV) center, as well as the nature of the second axial ligand, were varied to optimize the photoactivation rate of the Pt(IV) prodrug [64]. The linker between bodipy moiety and Pt(IV) center consisted of 2, 3, 4, 6, or 8 freely rotating CH_2_ groups. Moreover, as the second axial ligand, hydroxyl for Pt(IV) prodrugs **18**–**22** or acetyl for Pt(IV) prodrugs **17, 23**–**26** was used. Hence, ten Pt(IV) prodrugs **17**–**26** were reported (Figure 13). The stability of the Pt(IV) prodrugs **17**–**26** in the dark and the rate of reduction was monitored by HPLC. More than 46% of prodrugs **18**–**22** with hydroxide group were reduced within 6 h, while 86–93% of acetylated Pt(IV) prodrugs **17**, **23**–**26** remained stable after 24 h of incubation. Acetylated Pt(IV) prodrugs **17, 23**–**26** showed a higher activation rate than prodrugs with hydroxyl axial ligand **18**–**22**, with approximately 20 s of white light irradiation (2 mW/cm^2^) required until the full reduction of prodrugs **23**–**26**. For hydroxylated Pt(IV) prodrugs **18**–**22**, 8 min of irradiation were required for complete reduction. Thus, acetylated Pt(IV) prodrugs **17**, **23**–**26** were more stable in the absence of irradiation while being prone to light-induced reduction.

Interestingly, the Pt(IV) prodrug **23** with 3 freely rotating CH_2_ links showed the highest activation rate, while both Pt(IV) prodrugs with two freely rotating CH_2_ bonds **17** and **18** demonstrated the slowest reduction rate. The proposed explanation is that with the length of a chain 3 and longer, the BODIPY ligand is able to fold back to the Pt(IV) center, thereby facilitating the reduction rate.

The hypothesis was supported by observing the decrease of the Pt(IV) prodrugs **19** and **23** reduction rate in a more viscous solution containing 20% glycerol, which inhibits the free rotation of chemical bonds [79]. Without glycerol, 31.8% of prodrug **23** remained after 10 s of irradiation (white light, 2 mW/cm^2^), while 50.2% of prodrug **23** was intact in the presence of glycerol under the same irradiation dose. In contrast, the reduction rate of prodrugs **17** and **18** did not depend on the presence of glycerol. Thus, flexible linkers of Pt(IV) prodrugs **19** and **23** facilitate the photoreduction process by adopting the optimal conformation for electron transfer. At the same time, the short linker of Pt(IV) prodrugs **17** and **18** is too rigid to fold back to the Pt(IV) center.

MTT assay was utilized to evaluate the cytotoxicity of the Pt(IV) prodrugs **17**–**26** (Table 7). Complexes with long linkers **22**, **25,** and **26**, as well as complexes with the shortest linker **18**, were non-cytotoxic even under irradiation, while Pt(IV) prodrugs **23** and **19** showed increased cytotoxicity when irradiated. The IC_50_ values in the dark were higher than 100 µM for the Pt(IV) prodrug **23,** while under white light irradiation (2 mW/cm^2^, 30 min) the IC_50_ for both prodrugs were lower than 50 µM.

Thus, it was proven that the length of a linker between a Pt(IV) core and a photoactive ligand has a significant impact on the reduction rate of the Pt(IV) prodrug.

#### 2.2.7. Pt(IV) Prodrug Conjugated with Bodipy Derivative via Benzoic Acid Moiety in Meso-Position

**Figure 14:**
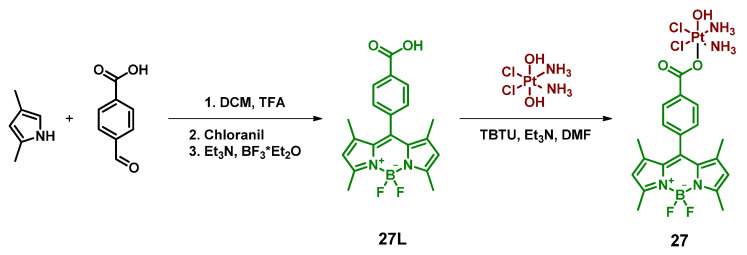
Synthesis of the Pt(IV) prodrug Oxoplatin-B **27** with bodipy derivative in axial position.

An alternative synthetic approach was used to conjugate bodipy moiety with Pt(IV) complex in a report by Bera et al. [65]. BODIPY ligand with benzoic acid in meso-position was conjugated with oxoplatin by activation with TBTU (Figure 14). Without the reducing agent in the dark, the degradation of the Pt(IV) prodrug **27** was negligible, while in the presence of 5 equivalents of ascorbic acid the complex showed a 50% reduction within 24 h of incubation.

The toxicity of the Pt(IV) prodrug **27** was low in the absence of irradiation and a 10–25-fold increase was observed after irradiation with white light (400–700 nm, 13 mW/cm^2^, 30 min) (Table 8). Notably, IC_50_ values on normal lung epithelial cell line HPL1D after irradiation were only 46 µM, 12–45 folds lower than on malignant MCF-7 and A-549 cell lines (IC_50_ values 3.8 and 1.1 µM, respectively). Cellular uptake of the Pt(IV) prodrug **27** was studied in comparison with the free ligand **27L** using FACS (fluorescence-activated cell sorting) analysis. The fluorescence intensity of A549 cells incubated with 5 µM of the Pt(IV) prodrug **27** was significantly higher compared to cells incubated with the free ligand **27L**, indicating the role of Pt core in axial ligand delivery to the cells.

The ability of the Pt(IV) prodrug **27** to generate ROS was studied using singlet oxygen trap 1,3-diphenylisobenzofuran (DPBF). Pt(IV) prodrug **27** was found to be a more efficient singlet oxygen generator than the free ligand **27L**. The singlet oxygen quantum yield for the Pt(IV) prodrug **27** and the free ligand **27L** was determined as 0.29 and 0.18, respectively. Thus, the Pt(IV) prodrug **27** was proven to be an efficient PDT agent, capable of generating ROS upon irradiation.

Incubation of the Pt(IV) prodrug **27** with mitochondria-staining dye revealed that the prodrug was almost quantitatively located within mitochondria. JC-1 assay, which allows determination of how mitochondria membrane potential is changed upon exposure to the drug, demonstrated a significant loss of mitochondrial membrane integrity when cells with the Pt(IV) prodrug **27** were irradiated, while the almost negligible effect was observed when cells were treated with the Pt(IV) prodrug **27** in the dark.

The ability of the Pt(IV) prodrug **27** to release cisplatin and the free axial ligand upon white light irradiation was studied using ^1^H NMR and ESI-MS. During 24 h of irradiation, the gradual disappearance of amine ligands signal in ^1^H NMR was observed, indicating the reduction of the Pt(IV) center. The ESI-MS analysis of the irradiated sample of the Pt(IV) prodrug showed the presence of cisplatin as well as the free ligand **27L**, which confirms that the prodrug releases Pt(II) complex and BODIPY upon irradiation. The ability of cisplatin to bind guanosine bases after release from the Pt(IV) prodrug **27** was studied by irradiating the solution of the Pt(IV) prodrug **27** and 9-ethylguanine (9-EtG) with white light (400–700 nm, 2.4 mW/cm^2^, 3 h). Subsequently, ^1^H NMR of the mixture showed a shift of the H8 proton of 9-EtG, indicating the binding of cisplatin at the N7 position.

#### 2.2.8. Pt(IV) Prodrug with Red-Light Absorbing Bodipy Derivative in Axial Position

In a subsequent report, Bera et al. used the same approach to functionalize cisplatin with red-light absorbing bodipy moiety [66]. Log *p* value 0.06 of the Pt(IV) prodrug **28** was considered optimal for accumulation in mitochondria and endoplasmic reticulum (ER). 

**Figure 15:**
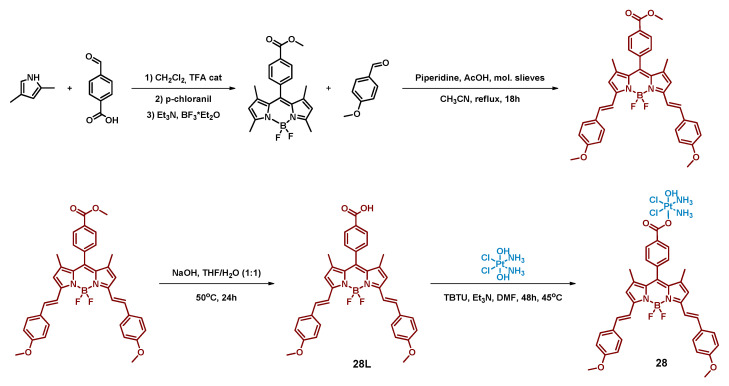
Synthesis of the Pt(IV) prodrug **28** with red-light absorbing bodipy derivative in axial position.

ER is an attractive target for cytotoxic agents as it is responsible for synthesis, folding, and transportation of cellular proteins, hence damage to its normal function could trigger apoptosis (Figure 15) [80].

The complex **28** and the ligand **28L** showed peak absorption in the red-light region 650 nm and quantum yield in DMSO was 0.45 and 0.37 for ligand and the complex, respectively. Stability of the Pt(IV) prodrug **28** in physiological conditions in the dark was examined via UV-Vis spectroscopy; no changes in the spectrum were observed within 48 h, indicating that the complex is stable. The complex was also found to be stable both at high (pH = 9) and low (pH = 3) pH values. In the presence of GSH, complex **28** was also stable for 24 h in the dark. Under red-light irradiation (642 nm, 100 mW/cm^2^) and in the presence of ascorbic acid, the ^1^H NMR peak of Pt(IV)-bound NH_3_ protons disappeared in 30 min.

The ability of the Pt(IV) prodrug **28** to generate singlet oxygen was investigated using singlet oxygen trap 1,3-diphenylisobenzofuran (DPBF). Irradiation of the solution of the Pt(IV) prodrug in the presence of DPBF with red light (642 nm, 100 mW/cm^2^) led to a decrease in DPBF absorption, indicating ROS formation.

The cytotoxicity of the Pt(IV) prodrug **28** was evaluated on malignant cell lines HeLa and MCF-7, and on normal lung epithelial cell line HPL1D (Table 9). Pt(IV) prodrug **28** was non-toxic in the dark, while under irradiation (600–720 nm, 30 J/cm^2^) the complex demonstrated submicromolar IC_50_ values. It is worth noting that even under irradiation, Pt(IV) prodrug **28** cytotoxicity on normal cell line HPL1D was more than 10-fold lower than on cancer cell lines.

To determine the intracellular distribution profile of the Pt(IV) prodrug **28**, colocalization assays were performed. The prodrug primarily accumulated in mitochondria and in the endoplasmic reticulum rather than in the nucleus, confirming that those organelles are the main target of the compound. Formation of ROS in prodrug **28**-treated cells under irradiation (600–720 nm, 30 J/cm^2^) was confirmed using 2′,7′-dichlorofluorescein diacetate (DCFDA), while no ROS formation was observed in the dark.

The ability of the Pt(IV) prodrug **28** to induce mitochondrial dysfunction in HeLa cells was evaluated using JC-1 assay, which showed a significant MMP decrease under irradiation (600–720 nm, 30 J/cm^2^).

Thus, the Pt(IV) prodrug **28** with red-light absorbing bodipy moiety in the axial position demonstrated the ability to release Pt(II) complex and generate ROS upon irradiation. It was established that the prodrug exhibits its toxicity by damaging mitochondria and the endoplasmic reticulum instead of the nucleus.

#### 2.2.9. Pt(IV) Prodrug with Two Cyanine-Based Ligands in Axial Position

**Figure 16:**
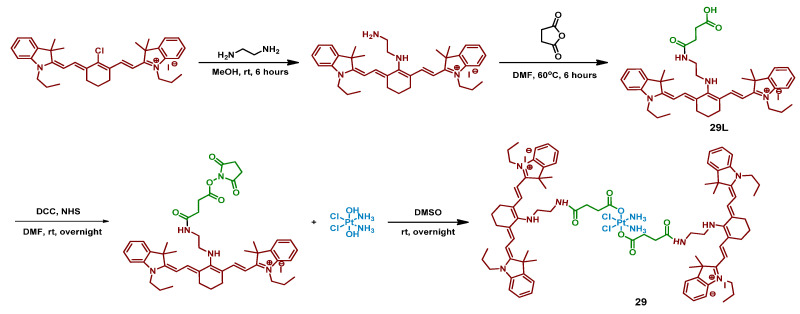
Synthesis of the Pt(IV) prodrug **29** with cyanine derivative in axial position.

Cyanine dyes are widely used as PDT agents with absorption peaks in NIR range [81]. Heptamethine cyanine dyes could also act as photocatalysts of several reactions and accumulate specifically in mitochondria [82]. Utilizing those properties of cyanine dyes, Li et al. synthesized a prodrug **29** of cisplatin with two moieties of cyanine photoactive ligand based on IR-780 dye (Figure 16) [67].

Stability of the Pt(IV) prodrug **29** was determined by UV-Vis spectroscopy. The complex was stable in the dark within 48 h, while about a third of the Pt(IV) prodrug **29** was reduced with an excessive amount of sodium ascorbate (50 equiv.), indicating overall good stability towards reduction. The photoreduction of the Pt(IV) prodrug was studied using X-ray photoelectron spectroscopy (XPS). When irradiated with 650 nm red light (10 mW/cm^2^, 30 min), the peaks in the XPS spectrum shifted to the lower energy values, thus indicating the photoreduction of the Pt(IV) prodrug **29** and release of cisplatin.

Considering that cyanine should facilitate accumulation of the Pt(IV) prodrug **29** in mitochondria, colocalization experiments were used to study intracellular distribution of Pt(IV) prodrug **29** in A549 and A549cisR cells. The results clearly showed that the Pt(IV) prodrug **29** was located mainly in mitochondria after 24 h of incubation. Subsequent ICP-MS assay demonstrated that Pt content in mitochondria was 4.1-folds higher than in the nucleus.

Cytotoxicity of the Pt(IV) prodrug **29** was evaluated on several cisplatin-sensitive and cisplatin-resistant cell lines, including MCF-7, MCF-7cisR, A549, A549cisR, A2780, and A2780cisR (Table 10). In the absence of light, the toxicity of the Pt(IV) prodrug **29** was close to that of cisplatin. In contrast, under irradiation (650 nm, 10 mW/cm^2^, 30 min), the toxicity of the Pt(IV) prodrug **29** increased 3-5-fold. It is worth noting that the toxicity of the Pt(IV) prodrug **29** was unaffected by the resistance of the cell lines, thus indicating its ability to overcome resistance to cisplatin.

The ability of the Pt(IV) prodrug **29** to induce cell death was evaluated by flow cytometry. The results showed that 82.7% of A549cisR cells treated with the Pt(IV) prodrug **29** and irradiated (650 nm, 10 mW/cm^2^, 30 min) were apoptotic or necrotic, compared to only 17% for cisplatin. Pt(IV) prodrug also demonstrated light-induced platination of DNA: 4.7 µg of Pt were detected after incubation in the dark, while 40.7 µg—almost 9-fold—were detected after irradiation. The ability of the Pt(IV) prodrug **29** to affect mitochondrial membrane potential (MMP) was investigated using the JC-1 assay. While cisplatin or the free ligand had little to no effect on MMP, the potential of cells treated with the Pt(IV) prodrug and irradiation was greatly reduced, indicating that the Pt(IV) prodrug **29** is toxic to mitochondria. 

To summarize, Pt(IV) prodrug **29** is a cisplatin prodrug with a photoactive heptamethine cyanine dye that can be reduced to Pt(II) drug under red-light irradiation (650 nm), overcome cisplatin resistance in cell lines, and accumulate in mitochondria, changing their membrane potential under irradiation. 

#### 2.2.10. Pt(IV) Prodrug-Functionalized Photoactive Polymer

**Figure 17:**
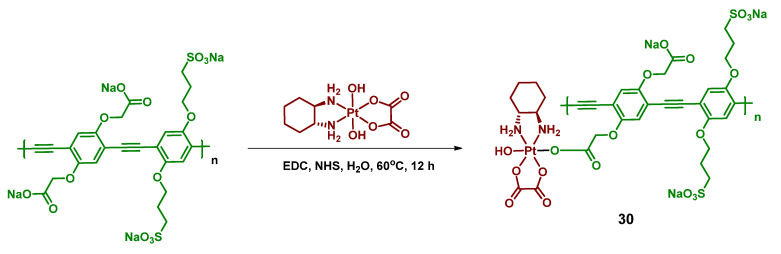
Synthesis of the polymer Pt(IV) prodrug **30** (exemplary monomer) with poly(phenylene ethynylene in axial position.

Conventional Pt(IV) prodrugs that are capable of activation under irradiation consist of a platinum core, its equatorial ligands, and axial ligands–light-sensitive small molecules. An alternative approach to the design of photoactivated Pt(IV) prodrugs was demonstrated by Sun et al. [68]. Poly(phenylene ethynylene) (PPE) was chosen as a light-absorbing scaffold due to its high molar absorptivity and good photostability [83,84]. Moreover, due to the sulfonate (SO_3_^−^) and carboxylate (CO_2_^−^) moieties in the structure, the PPE polymer shows great water-solubilizing features. The Pt(IV) prodrug **30** was synthesized by activating the carboxylate fragments by EDC and NHS, followed by a reaction with oxo-oxaliplatin (Figure 17). The resulting Pt(IV) polymer prodrug **30** was water soluble at room temperature.

The rate of photolysis of prodrug **30** was studied in the presence or absence of sodium ascorbate using UV-Vis absorption spectra. In both cases, within 120 min of irradiation (400 nm, 5 mW/cm^2^) the long-wavelength shoulder at 443 nm disappeared, indicating a release of oxaliplatin from the polymer. Using dynamic light scattering, the change of diameter of the Pt(IV) prodrug 30 aggregates was assessed under blue-light irradiation. Within 60 min, the diameter of aggregates decreased from 260 to 160 nm, which also indicated the photolysis of the polymer prodrug **30**.

The rate of the prodrug **30** photolysis was quantified using HPLC. After 24 h in the dark, only 8% of oxaliplatin was released. In contrast, under blue-light irradiation (400 nm, 5 mW/cm^2^, 30 min) the yield of oxaliplatin released from the Pt(IV) prodrug was 60% without NaAsc and 90% with NaAsc after 30-min-exposure to light. For PPE polymers, the significant two-photon absorption cross-section in the 700–800 nm range [84,85] was previously shown. To test whether the prodrug **30** could release oxaliplatin after two-photon absorption, the solution of the Pt(IV) prodrug **30** was irradiated with 100 fs pulses (725 nm, 800 mW/cm^2^, 1 h). Approximately 50% of oxaliplatin was released, thus showing the possibility of photorelease of oxaliplatin from prodrug **30** under near-infrared irradiation.

To determine the mechanism of the Pt(IV) prodrug **30** photolysis, a series of photophysical experiments were conducted. The fluorescence quantum yield of prodrug **30,** as well as its fluorescence lifetime, was significantly lower than for PPE (Table 11). This indicates the quenching of fluorescence due to photoinduced electron transfer.

The picosecond transient absorption measurements conducted for PPE and prodrug **30** revealed that the transient state of prodrug **30** decays significantly faster than free polymer PPE (Table 12). More than 80% of the prodrug **30** TA decays in less than 10 ps, while two TA components have lifetimes longer than 30 ps. The authors suggested that the main component of the prodrug **30** TA spectrum is the absorption of the polymer radical cation (PPE*+) [86,87], produced by the ultrafast electron transfer from the polymer to the Pt(IV) center.

**Figure 18:**
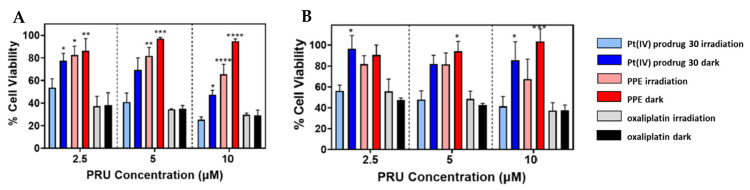
SKOV-3 cells viability, incubated with the Pt(IV) prodrug **30**, PPE, and oxaliplatin under irradiation (20 min, 460 nm, 7 mW/cm^2^) or in the dark. (**A**) 24 h of incubation before irradiation, no removal of residual compound prior to the irradiation. (**B**) 1 h of incubation before removal of residual compounds and irradiation in fresh media. **** *p* < 0.0001, *** *p* = 0.0003, ** *p* < 0.01, and * *p* < 0.05. Reproduced with permission from Ref. [64]. Copyright 2022 American Chemical Society.

To evaluate the photoinduced cytotoxicity of PPE against malignant cell types, SKOV-3 ovarian cancer cells were chosen as a model cell line and were treated with PPE, then irradiated with 460 nm light (Figure 18A). Pt(IV) prodrug **30** showed high activity under irradiation comparable to oxaliplatin and it was significantly higher than for PPE. However, at high concentrations, PPE showed some cytotoxic effect, probably from ROS generation. To determine whether the observed effect was due to the polymer activation in the extracellular medium, a washing step was added before the irradiation to remove the compounds from the extracellular medium (Figure 18B). The activity of the Pt(IV) prodrug **30**, as well as of PPE, was decreased, indicating the contribution of activation of the polymer in the extracellular medium. Thus, the Pt(IV) prodrug **30** was established as a light-activated Pt(IV) prodrug.

Immunofluorescent assay of DNA damage marker γH2A.X demonstrated the similar nuclear intensity of prodrug **30** under light irradiation and the equivalent amount of oxaliplatin, proving that the main cytotoxicity mechanism of PPE-Pt is DNA damage induced by photoreleased oxaliplatin. Thus, Pt(IV) prodrug **30** is a photocaged polymer prodrug that could release cytotoxic oxaliplatin via one-photon and two-photon absorption. Upon irradiation, the released Pt(II) complex damages the DNA, exercising the same mechanism of cytotoxicity as oxaliplatin.

## 3. Conclusions

The combination of Pt(IV) prodrugs with photoabsorbing molecules allows precise control over their cytotoxic activity in space and time. Such Pt(IV) prodrugs are almost non-toxic in the absence of irradiation, but by applying light specifically to a designated area, a cytotoxic effect could be induced in the tumor area without damaging normal tissues. Moreover, due to most photoabsorbers for Pt(IV) photoactivation being efficient PDT agents, the resulting Pt(IV) complexes could act as dual-action prodrugs, releasing Pt(II) cytostatics and generating toxic ROS under light irradiation.

A mixture of Pt(IV) prodrugs with riboflavin derivatives Rf, TARF, Lf, and FMN or flavoproteins as photocatalysts was proven to be an effective PACT agent. Mixtures were non-toxic in the dark, but under low-dose irradiation released Pt(II) species. Mechanistic studies showed that Pt(IV) prodrugs reduction occurs through riboflavin reduced form RfH^−^ forming an H-bonded intermediate with a Pt(IV) complex. The combinational approach also proved its effectiveness when FMN was loaded into TACN-modified Au nanoparticles. Furthermore, Salassa et al. showed that the catalytical activity of flavoproteins towards Pt(IV) prodrugs reduction could be controlled by site-targeted mutagenesis.

Photoabsorbers with different maximum absorption wavelengths were used to obtain Pt(IV) prodrugs with controllable photoactivation. Many of the ligands utilized, including coumarin, bodipy derivatives, and PPE, had absorption in the range 400–500 nm; slightly more red-shifted ligand was rhodamine B with absorption at around 570 nm. Pyropheophorbide a, bodipy-based ligand **28L**, and cyanine-based ligand **29L** were the most red-shifted among ligands used in the discussed reports, with absorption maximums at 640–660 nm. Moreover, by loading Phorbiplatin prodrug derivative **7** onto nanocrystals capable of generating upconversion luminescence, even more red-shifted nanoprodrug with an absorption maximum at 808 nm was obtained. It is also worth noting that for polymer Pt(IV) prodrug **30**, photoinduced release is possible after two-photon absorption at 725 nm. Bodipy moiety turned out to be the most frequently utilized as a photoactive ligand for Pt(IV) prodrugs **17**–**28**. Prodrugs **17**–**26** were conjugated through a linker in the side aliphatic moiety of a pyrrole scaffold. In contrast, Pt(IV) prodrugs **27** and **28** were designed with a benzoic acid scaffold in the *meso*-position of the corresponding ligands **27L** and **28L**. Moreover, bodipy-based ligands showed absorption in green light (ca. 500 nm) for Pt(IV) prodrugs **17**–**27**, as well as in red light (642 nm) for the Pt(IV) prodrug **28**. Those reports illustrate the synthetic versatility of bodipy-based photoabsorbers that could be used as a ligand for light-activated Pt(IV)prodrugs.

All photocaged Pt(IV) prodrugs showed a significant increase in toxicity under irradiation compared to the Pt(II) counterparts. Coumaplatin **14** with the vector peptide R_8_K showed a 26-fold increase in toxicity on the A2780 cell line. The most drastic increase in vitro was observed for red-light absorbing Phorbiplatin **6**, with a 976-fold increase in toxicity on the A2780cisR line. Toxicity of the red-light absorbing Pt(IV) prodrug **28** on selected cell lines increased more than 100-fold. It should also be noted that all Pt(IV) prodrugs discussed demonstrated high IC_50_ values on normal cell lines in the dark, thus proving the selectivity of photosensitive Pt(IV) prodrugs. Interestingly, for many photocaged prodrugs, a specificity towards mitochondria was reported. Rhodaplatin **16**, bodipy-based Pt(IV) prodrugs **27** and **28**, and cyanine-based Pt(IV) prodrug **29** accumulated primarily in mitochondria, hence damage to mitochondria was the main toxicity mechanism of those prodrugs.

Various mechanisms of Pt(IV) prodrugs photoreduction were suggested. For Phorbiplatin **8** and Rhodaplatins **15** and **16,** it was shown that a reductant, namely, ascorbic acid is required to transfer an electron and reduce the ligand from the excited state. From the reduced ligand, the electron is then transferred to the Pt(IV) core. In contrast, for Coumaplatin **14** and its precursor Pt(IV) prodrug **13**, it was proven that no specific reductant is required and the complex is reduced under irradiation by water. For polymer Pt(IV) prodrug **30**, the initial electron transfer step from PPE to Pt(IV) requires no reductant; however, the resulting PPE^+*^ could then be regenerated by sodium ascorbate.

Several photosensitive Pt(IV) prodrugs were tested in vivo. Phorbiplatin **8** and nanoprodrug **12** showed striking antitumor efficiency. For Phorbiplatin **8**, the reduction of tumor weight by 62% compared to the control group was observed after 12 days of therapy. Nanoprodrug **10** with erythrocyte-binding peptide vector demonstrated even more striking results. A more than 100-fold decrease in tumor volume in comparison with the control was observed in mice treated with nanoprodrug **12**; moreover, 2 of the 5 mice were tumor-free after 14 days of therapy.

To summarize, a combination of the Pt(IV) prodrug approach with photosensitive organic molecules results in antitumor agents with a controllable mode of action. Such prodrugs are non-toxic towards normal cells that are not irradiated, while they are simultaneously highly efficient against malignant cells in vitro and in vivo in the presence of irradiation. Light-controlled temporal and spatial selectivity opens the path for a new generation of Pt-containing antitumor drugs that exert their cytotoxic action precisely where required. Combined with drug delivery vehicles, i.e., tumor-targeting vectors such as cell-penetrating and erythrocyte-binding peptides, Pt(IV) prodrugs might become the “magic bullet” for cancer proposed by Ehrlich [88].

## Figures and Tables

**Table 1:** Publications dedicated to the photocatalytic reduction of Pt(IV) prodrugs.

Pt(IV) Prodrugs Used in Paper	Flavines	Electron-Donor	Results	Reference
**1**	Riboflavin (Rf)	2-(*N*-morpholino)ethanesulfonic acid (MES)	Proven that riboflavin might reduce Pt(IV) prodrug **1** in presence of a reducing agent.	[51]
**1**, **2**, **4**	Flavin adenine dinucleotide (FAD), mini Singlet Oxygen Generator (miniSOG), NAD-H oxidase (NOX), Glutathione reductase (GR)	MES, NAD-H	Demonstrated that activation of Pt(IV) prodrugs **1**, **2**, and **4** may be induced by flavoproteins.	[52]
**1**, **4**	Riboflavin	MES	Demonstrated that mixtures Rf-1 and Rf-4 are active toward the PDT-resistant Capan-1 cell line.	[53]
**1**	FMN incorporated in TACN-modified Au nanoparticles	TACN-modified Au nanoparticles	A system that contains TACN-modified Au nanoparticles and incorporated FMN was developed, which may cause photocatalytic reduction of Pt(IV) prodrug **1** without other reducing agents.	[54]
**1**, **3**, **4**, **5**	Rf, FMN, Tetraacetylriboflavin (TARF), Lumiflavin (Lf), miniSOG	NAD-H	Defined photocatalytic properties of other riboflavin derivatives and photocatalytic mechanism proved by flash-photolysis.	[55]
**1**, **3**	miniSOG and its mutants Q103V, Q50E, Q50W	NAD-H	The photocatalytic ability of several mutant miniSOG proteins was defined.	[56]

**Table 2:** Photosensitive Pt(IV) prodrugs, classes of photoabsorbers used, fold increase (FI) on selected cell lines reported, wavelength and light doses applied in cytotoxicity tests.

№/Name	Prodrug Pt(IV) Structure	Nature of Axial Moiety	Fold Increase: IC_50_ of Pt(II) Drug Under Irradiation/IC_50_ of Pt(IV) Prodrug Under Irradiation	Irradiation, Wavelength λ (nm), and Dosage (Irradiation Power and Period)	Reference
**8** Phorbiplatin	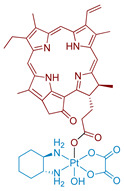	Porphyrin	974 (A2780cisR)	650 nm, 6.3 J/cm^2^ (7 mW/cm^2^, 15 min)	[60]
**9** Phorbiplatin-COOH	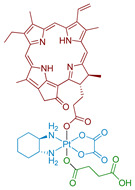	Porphyrin	-	-	[61]
**10**	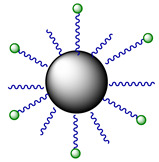	Nanocrystals, modified with **9**	≈16 (A2780cisR)	808 nm, 150 J/cm^2^ (500 mW/cm^2^, 5 min)	[61]
**11**	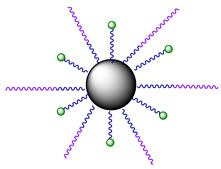	Nanocrystals, modified with **9** and PEG	-	-	[61]
**12**	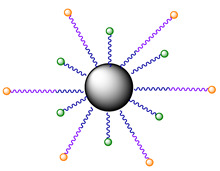	Nanocrystals, modified with **9** and ERY_1_ peptide	-	In Vivo: 808 nm, 900 J/cm^2^ (500 mW/cm^2^, 30 min) per round of irradiation	[61]
**13**	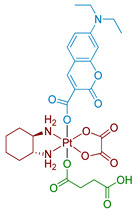	Coumarin	>2 (A2780cisR)	450 nm, 28.8 J/cm^2^ (8 mW/cm^2^)	[62]
**14** Coumaplatin	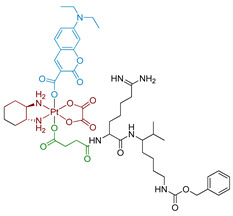	Coumarin	26 (A2780)	450 nm, 28.8 J/cm^2^ (8 mW/cm^2^)	[62]
**15** Rhodaplatin	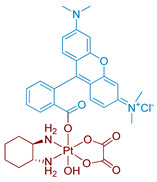	Rhodamine B	9.8 (A2780cisR)	400–760 nm, 7.2 J/cm^2^ (4 mW/cm^2^, 30 min)	[58]
**16** Rhodaplatin	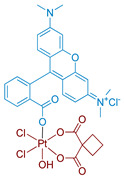	Rhodamine B	9.8 (A2780cisR)	400–760 nm, 7.2 J/cm^2^ (4 mW/cm^2^, 30 min)	[58]
**17** Bodi-Pt	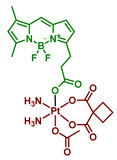	Bodipy	7.2 (A2780)	≈490 nm, 23.4 J/cm^2^ (13 mW/cm^2^, 30 min)	[63]
**18**	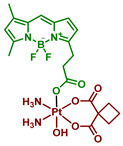	Bodipy	>>2 (A2780)	400–760 nm, 3.6 J/cm^2^ (2 mW/cm^2^, 30 min)	[64]
**19–22**	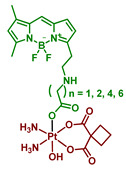	Bodipy	>>2 (A2780)	400–760 nm, 3.6 J/cm^2^ (2 mW/cm^2^, 30 min)	[64]
**23–26**	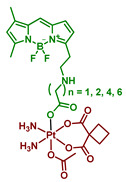	Bodipy	>>2 (A2780)	400–760 nm, 3.6 J/cm^2^ (2 mW/cm^2^, 30 min)	[64]
**27** Oxoplatin B	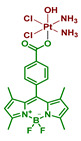	Bodipy	33 (HeLa)	400–700 nm, 10 J/cm^2^ (13 mW/cm^2^, 30 min)	[65]
**28**	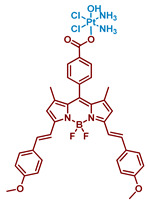	Bodipy	117 (HeLa)	600–720 nm, 30 J/cm^2^	[66]
**29**	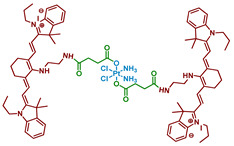	Heptamethine cyanine	>4 (A2780cisR)	650 nm, 18 J/cm^2^ (10 mW/cm2, 30 min)	[67]
**30**	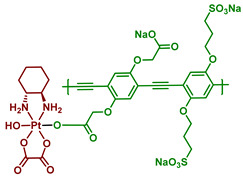	Poly(phenylene ethynylene)		460, 8.4 J/cm^2^ (7 mW/cm^2^, 20 min)	[68]

**Table 3:** Cytotoxicity of Phorbiplatin **8** in comparison with free pyropheophorbide a and Oxaliplatin, with or without light irradiation (650 nm, 7 mV/cm^2^, 15 min) on cell lines A2780 (ovarian cancer), A2780cisR (cisplatin-resistant ovarian cancer), MCF- 7 (breast cancer), 4T1 (xenograft mouse breast tumor), and MRC-5 (embryonic fibroblasts). FI—index determined by the ratio of toxicity of oxaliplatin and phorbiplatin.

Cell line	IC_50_ [µM]						FI
	Oxaliplatin		Pyropheophorbide a (PPA)		Phorbiplatin 8		
	Light	Dark	Light	Dark	Light	Dark	
A2780	68 ± 9	76 ± 4	0.34 ± 0.05	>10	0.13 ± 0.01	>10	523
A2780cisR	185 ± 8	162 ± 9	0.23 ± 0.01	>10	0.19 ± 0.01	>10	974
MCF-7	78.6 ± 8.7	110 ± 4.3	0.20 ± 0.02	>10	0.044 ± 0.004	>10	1786
4T1	7.6 ± 1.3	8.7 ± 0.9	0.16 ± 0.02	>10	0.13 ± 0.004	>10	58
MRC-5		122 ± 5.2		>10		>10	

**Table 4:** Antiproliferative activity of Coumaplatin 14, complex 13, and oxaliplatin on HCT116 p53^+/+^ cell lines (colon cancer cell line with p53 expression), HCT116 p53^−/−^ (colon cancer cell line without p53 expression), HT29 (colon cancer), HeLa (cervical cancer), MCF-7 (breast adenocarcinoma), MDA-MB-231 (triple negative breast cancer), A2780 (ovarian cancer), A2780cisR (cisplatin-resistant ovarian cancer cell line), A549 (lung cancer), A549cisR (cisplatin-resistant lung cancer cell line), and MRC-5 (embryonic fibroblasts) in the dark and under irradiation (450 nm, 8 mW/cm^2^, 1 h).

Cell Line	IC_50_ (µM)							
	Oxaliplatin		Pt(IV) Prodrug 13		Coumaplatin 14		PI	FI
	Light	Dark	Light	Dark	Light	Dark		
HCT116 p53^+/+^	29.3 ± 3.2	31.6 ± 5.2	52.8 ± 6.6	>100	0.9 ± 0.2	47.1 ± 5.9	52	32
HCT116 p53^−/−^	124.9 ± 9.7	127.2 ± 11.4	89.3 ± 5.6	>100	1.3 ± 0.3	80.6 ± 7.1	62	96
HT29	37.8 ± 3.1	41.2 ± 2.7	81.5 ± 5.5	>100	2.7 ± 0.4	51.6 ± 4.2	19	14
HeLa	34.1 ± 3.3	38.2 ± 2.4	61.8 ± 4.8	>100	5.6 ± 1.1	38.8 ± 4.2	7	6
MCF-7	44.1 ± 7.7	46.5 ± 8.1	86.2 ± 93	>100	5.5 ± 2.1	52.1 ± 6.9	10	8
MDA-MB-231	40.5 ± 5.61	42.3 ± 7.5	79.4 ± 6.6	>100	8.1 ± 3.0	69.3 ± 5.2	9	5
A2780	25.2 ± 4.7	26.6 ± 4.1	56.2 ± 5.6	>100	3.9 ± 0.6	42.1 ± 4.7	11	6
A2780cisR	127.4 ± 8.7	132.8 ± 9.2	>100	>100	4.9 ± 0.8	72.6 ± 4.8	15	26
A549	61.9 ± 7.0	64.4 ± 6.1	81.3 ± 6.7	>100	6.9 ± 1.1	49.2 ± 4.2	7	9
A549cisR	172 ± 6.9	175.7 ± 10.2	>100	>100	4 ± 0.5	114.8 ± 7.7	29	43
MRC-5		59.7 ± 7.1		>100		93.9 ± 6.8	–	–

**Table 5:** Antiproliferative activity of Rhodaplatin 15 and 16 compared to carboplatin and oxaliplatin, respectively, on A2780 (ovarian carcinoma), A2780cisR (cisplatin-resistant ovarian carcinoma), MCF-7 (breast cancer, A549 (lung carcinoma), A549cisR (cisplatin-resistant lung carcinoma), HCT116 (colorectal cancer), and MCR5 (fetal lung normal cell line) cell lines in the dark and under white light irradiation (400–760 nm, 4 mW/cm^2^, 30 min).

Cell Line	IC_50_ [µM]						IC_50_ [µM]					
	Carboplatin		Rhodaplatin 15		PI	FI	Oxaliplatin		Rhodaplatin 16		PI	FI
	Light	Dark	Light	Dark			Light	Dark	Light	Dark		
A2780	322 ± 33	301 ± 28	44 ± 5	220 ± 17	5	7.3	64 ± 6	68 ± 6	25 ± 2	108 ± 9	4.4	2.6
A2780cisR (RF)	>400 (—)	>400	41 ± 5 (0.9)	250 ± 18	6.1	>9.8	199 ± 21	187 ± 19	20 ± 7 (0.8)	136 ± 13	6.7	9.8
MCF-7	>400	>400	77 ± 9	245 ± 17	3.2	>5.2	103 ± 12	113 ± 13	43 ± 3	133 ± 11	3.1	2.4
A549	>400	>400	57 ± 5	251 ± 15	4.4	>7.0	87 ± 6	95 ± 12	29 ± 5	104 ± 15	3.7	3.1
A549cisR (RF)	>400 (—)	>400	61 ± 6 (1.1)	289 ± 17	4.7	>6.5	218 ± 10 (2.5)	212 ± 9	33 ± 5 (1.1)	142 ± 10	4	5
HCT116	>400	>400	47 ± 5	248 ± 18	5.3	>10.7	60 ± 4	58 ± 4	18 ± 1	112 ± 6	6.3	3.4
MRC-5		>400	>300		—	—		82 ± 7		116 ± 7	—	—

**Table 6:** IC_50_ values of Bodi-Pt **17** and carboplatin on MCF-7, MDA-MB-231, SKOV3, A2780, HeLa, A549, and WI-38 (fibroblast-like fetal lung) cell lines under dark (light (-)) and under green light irradiation (13 mW/cm^2^, 30 min, green light).

Cell Line	IC_50_ [µM]						
	BODI-Pt 17			Carboplatin		PI	FI
	Light	Dark	PI	Light	Dark		
MCF-7	15.7 ± 1.0	173.4 ± 8.8	11	642.6 ± 51.4	608.1 ± 61.0	0.9	38.7
MDA-MB-231	19.1 ± 4.7	162 ± 8.4	8.5	527.7 ± 54.6	498.3 ± 28.9	0.9	26.1
SKOV3	22.4 ± 0.8	>200	>8.9	991.7 ± 62.3	962.5 ± 110.7	1	43
A2780	31.0 ± 7.1	68.5 ± 5.0	2.2	254.0 ± 31.0	222.6 ± 11.8	0.9	7.2
HeLa	37 ± 1.5	>200	>5.4	600.1 ± 38.1	799.8 ± 33.0	1.3	21.6
A549	60.5 ± 14	148.9 ± 17.3	2.5	418.6 ± 75.0	395.9 ± 69.2	0.9	6.5
WI-38		>200			432.7 ± 35.7		

**Table 7:** IC50 values of Pt(IV) prodrugs **17–26** on the A2780 cell line in the dark and under irradiation (white light, 2 mW/cm^2^, 30 min).

Compounds	IC_50_ [µM]	
	A2780	
	Light	Dark
**18**	>100	>100
**19**	43.9 ± 5.2	69.8 ± 4.9
**20**	48.3 ± 2.0	74.8 ± 8.2
**21**	76.8 ± 9.9	>100
**22**	>100	>100
**17**	66.6 ± 7.0	>100
**23**	43.8 ± 3.9	>100
**24**	88.6 ± 15.7	>100
**25**	>100	>100
**26**	>100	>100

**Table 8:** IC50 values of Pt(IV) prodrug **27**, ligand **27L**, and cisplatin on MCF-7 (breast cancer), HeLa (cervical cancer), A549 (lung carcinoma), and HPL1D (human peripheral lung epithelial) cell lines under white light irradiation (400–700 nm, 13 mW/cm^2^, 30 min).

	IC_50_ [µM]						
Cell Line	Pt(IV) Prodrug 27		27L		Cisplatin		
	Light	Dark	Light	Dark	Light	Dark	FI
MCF-7	3.8 ± 0.4	>50	33.4 ± 1.1	>50	-	28 ± 3	7.4
HeLa	2.1 ± 0.1	>50	31 ± 1	>50	-	71 ± 3	33.8
A549	1.1 ± 0.3	>50	33 ± 1	>50	-	-	
HPL1D	46 ± 1	>50	>50	>50	-	-	

**Table 9:** Antiproliferative activity of Pt(IV) prodrug **28,** free ligand **28,** and cisplatin HeLa, MCF-7 and HPL1D (lung epithelial) cell lines in the dark and under irradiation (600–720 nm, 30 J/cm^2^).

Cell Line	IC50 [µM]							
	Pt(IV) Prodrug 28			28L			Cisplatin	
	Light	Dark	PI	Light	Dark	PI	Light	Dark
HeLa	0.58 ± 0.02	>100	>172	6.3 ± 0.1	>100	>15	68.7 ± 3.4	71.3 ± 2.9
MCF-7	0.76 ± 0.05	>100	>131	4.4 ± 0.4	>100	>23	—	69.7 ± 1.2
HPL1D	9.4 ± 0.6	>100	>11	37.6 ± 2.2	>100	>2		

**Table 10:** Antiproliferative activity of free ligand **29L**, cisplatin, mixture of cisplatin and **29L**, and Pt(IV) prodrug **29** on MCF-7, MCF-7cisR, A2780, A2780cisR, A549, and A549cisR in the dark and under irradiation (650 nm, 10 mW/cm^2^, 30 min).

	IC_50_ [µM]							
Cell Line	29L		Cisplatin		29L + Cisplatin		Pt(IV) Prodrug 29	
	Dark	Light	Dark	Light	Dark	Light	Dark	Light
MCF-7	>50	29.3	35.3	32.6	25	17.1	35.3	7.7
MCF-7cisR	>50	44.3	>50	>50	>50	31	44.9	8.9
A2780	>50	27.4	29.9	25.2	16.4	9.7	38	12
A2780cisR	>50	33.2	>50	>50	>50	29.6	44.6	12.5
A549	>50	27.7	49.7	43.3	35.5	19.7	38.6	11.1
A549cisR	>50	33.8	>50	>50	>50	29.1	>50	13.8

**Table 11:** Fluorescence Lifetimes and Quantum Yields of the Pt(IV) prodrug 30 and PPE in water.

	τ1, ps (α1)	τ2, ps (α2)	τ3, ps (α3)	Φf
PPE	160 (0.89)	380 (0.11)		0.19
Pt(IV) prodrug 30	17 (0.96)	220 (0.04)	920 (0.01)	0.013

**Table 12:** Picosecond transient absorption kinetics of the Pt(IV) prodrug 30 and PPE in water.

	τ1, ps (α1)	τ2, ps (α2)	τ3, ps (α3)
PPE	1.7 (0.43)	30 (0.29)	245 (0.28)
Pt(IV) prodrug 30	0.63 (0.54)	9.6 (0.31)	350 (0.15)

## Data Availability

Not applicable.

## Abbreviations

Bodipy, Boron dipyrromethene; CD, Circular dichroism; ct-DNA, calf thymus DNA; DCFDA, 2′,7′-dichlorofluorescein diacetate; DFT, Density functional theory; DPBF, 1,3-diphenylisobenzofuran; EDC, 1-Ethyl-3-carbodiimide; ER, endoplasmic reticulum; ERY1, glycophorin A-binding peptide; FAD, Flavin adenine dinucleotide; FDA, Food&Drug Administration; FI, Fold Increase: IC50 of Pt(II) drug under irradiation/IC50 of Pt(IV) prodrug under irradiation; FMN, Flavin mononucleotide; GOX, Glucose oxidase; GR, Glutathione reductase; ICP-OES, Inductively coupled plasma—optical emission spectroscopy; MAL, Maleimido; MALDI-TOF, Matrix-assisted laser desorption/ionization—time of flight; MES, 2-(N-morpholino)ethanesulfonic acid; miniSOG, mini Singlet Oxygen Generator; MTT, 3-(4,5-Dimethylthiazol-2-Yl)-2,5-Diphenyltetrazolium Bromide; NAD-H, Nicotinamide adenine dinucleotide; NCs, NaYbF4:Er@NaYF4:Yb/Nd@NaYF4:Ca nanocrystals; NHS, N-hydroxysuccinimide; NIR, Near-infrared; NOX, NADH oxidase; ODN, Oligodeoxynucleotide; PACT, Photo-activated chemotherapy; PBS, Phosphate saline buffer; PDT, Photodynamic therapy; PEG, Polyethylene glycol; PI, phototoxicity index: IC_50_ of Pt(IV) in the dark/IC_59_ of Pt(IV) prodrug under irradiation; PPA, Pyropheophorbide a; PPE, Poly(phenylene ethynylene); RhB, Rhpdamine B; ROS, Reactive oxygen species; RP-HPLC, Reverse-phase high-performance liquid chromatography; TA, Transient absorption; TACN, triazacyclononane; TARF, Tetraacetyl riboflavin; TBTU, 2-(1H-Benzotriazole-1-yl)-1,1,3,3-tetramethylaminium tetrafluoroborate; TCSPC, Time-correlated single-photon counting; UV-Vis, Ultraviolet-visible; XPS, X-ray photoelectron spectroscopy.

## Glossary

4T1, mammary breast cancer; A2780, ovarian cancer cell line; A2780cisR, cisplatin-resistant ovarian cancer; A549, lung adenocarcinoma; A549cisR, cisplatin-resistant lung adenocarcinoma; CAPAN-1, pancreatic adenocarcinoma; HCT116, colon cancer; HeLa, cervical cancer; HPL1D, lung epithelial; HT29, colon cancer; MCF-7, breast adenocarcinoma; MCF-7cisR, cisplatin-resistant, breast adenocarcinoma; MDA-MB-231, triple negative breast adenocarcinoma; MRC-5, lung fibroblasts; PC-3, prostate cancer; SKOV3, ovarian adenocarcinoma; WI-38, lung fibroblasts.
